# Retrospective comparison of side effects and outcomes of three-dimensional conformal vs. intensity-modulated radiation therapy in the palliative-intent treatment (4 Gy × 5 daily fractions) of canine intranasal tumors (2010–2017)

**DOI:** 10.3389/fvets.2023.1011949

**Published:** 2023-03-14

**Authors:** Dah-Renn Fu, Hsin-Yi Weng, Carlos Roberto Mendez Valenzuela, Isabelle F. Vanhaezebrouck, Jeannie M. Plantenga

**Affiliations:** ^1^Department of Veterinary Clinical Sciences, College of Veterinary Medicine, The Ohio State University, Columbus, OH, United States; ^2^Department of Comparative Pathobiology, College of Veterinary Medicine, Purdue University, West Lafayette, IN, United States; ^3^Department of Veterinary Clinical Sciences, College of Veterinary Medicine, Purdue University, West Lafayette, IN, United States

**Keywords:** 3D conformal radiotherapy, dog, intensity-modulated radiation therapy (IMRT), intranasal neoplasm, palliation, radiation therapy

## Abstract

**Objective:**

To compare the occurrence of radiation side effects and treatment outcomes in dogs with intranasal tumors treated with a total dose of 20 Gy delivered in 5 daily 4 Gy fractions using computer-based 3D conformal (3DCRT) or intensity-modulated (IMRT) radiation therapy plans.

**Design:**

Retrospective case series.

**Materials and methods:**

Medical records for dogs with intranasal tumors treated with 4 Gy × 5 fractions between 2010 and 2017 were reviewed. Radiation side effects, time to local progression (TTLP), progression-free survival (PFS) and survival time (OS) were evaluated.

**Results:**

Thirty-six dogs (24 carcinomas, 10 sarcomas and 2 others) met the inclusion criteria. Sixteen were treated with 3DCRT and 20 with IMRT. Clinical signs improvement or resolution were reported in 84% of dogs. The median time to clinical signs improvement was 12 days (1–88 days) after the end of treatment. Eight dogs treated with 3DCRT (8/16, 50%) and 5 with IMRT (5/20, 25%) were documented acute radiation side effects. Almost all were classified as grade 1 skin, oral or ocular acute side effects. Only one dog in 3DCRT group was demonstrated grade 2 skin acute effects. The median TTLP for dogs treated with 3DCRT or IMRT was 238 days and 179 days, respectively (*p* = 0.967). The median PFS for 3DCRT or IMRT was 228 days and 175 days, respectively (*p* = 0.940). The median OS for 3DCRT or IMRT was 295 days and 312 days, respectively (*p* = 0.787). No significantly differences were observed in side effects, TTLP, PFS and OS between 3DCRT and IMRT groups.

**Conclusions:**

Palliative-intent conformal radiation therapy given in five daily 4 Gy fractions relieved clinical signs with minimal radiation side effects, with no statistical difference in occurrence between 3DCRT and IMRT dogs.

## Introduction

Intranasal tumors account for 1–2% of all neoplasms in dogs (1). Nearly all canine intranasal tumors are malignant. Sixty to 75% are carcinomas and the bulk of the remainder are sarcomas. They are locally aggressive and invade surrounding tissues, but gross metastatic disease is not commonly identified on routine staging tests performed at the time of diagnosis (2–5). Dogs with intranasal tumors that remain untreated have a median overall survival time (OS) of 1.5–3.1 months (5, 6). Surgical resection is associated with significant morbidity and is rarely complete due to the complexity of the surrounding anatomical structures. Radiation therapy is a relatively effective and noninvasive treatment for local control of canine intranasal tumors (1–4, 6, 7).

Palliative care has been a mainstay of the treatment of human patients with advanced and incurable cancers for many years. Palliation is treatment to shrink solid masses, slow tumor growth, or control cancer symptoms (8–10). The goal of palliative-intent radiation therapy for dogs with intranasal tumors is to improve quality of life by relieving pain associated with bony lysis, inflammation, or tissue compression caused by tumors, and associated clinical signs, primarily epistaxis, nasal congestion and discharge, while minimizing acute radiation side effects and time under treatment (11–13). Several reports of palliative-intent radiation therapy for canine intranasal tumors have been published, but there is no standard palliative-intent protocol. Hypofractionation with larger fractional doses is commonly being used (11–14). The reported median OSs of dogs with intranasal tumors treated with palliative-intent hypofractionated radiation therapy ranges from 4.8 to 10.3 months (11–14).

A prospective study of 18 intranasal tumor dogs treated with a 4 Gy × 5 daily palliative-intent radiation protocol was published (13). Seventeen out of 18 (94.5%) dogs achieved clinical response. The median OS was 309 days and the median progression-free survival (PFS) was 178 days. Ten treatment plans were designed based on computed tomography (CT), two were magnetic resonance imaging (MRI) based, and six were manual planning. Half of all dogs experienced grade 1 to 2 acute radiation side effects in the study. However, the relationship between occurrence of side effects and type of plan has not been mentioned (13).

Radiation side effects can increase patients' discomfort and decrease their quality of life. The common radiation side effects for dogs with intranasal tumors treated with radiation therapy include alopecia, desquamation dermatitis, skin erythema, ocular discharge, conjunctivitis, decreased tear production, and oral mucositis (7, 11–14). Since the intranasal cavity is surrounded by critical structures, including the eyes, lenses, lacrimal glands and brain, it is important to avoid these critical organs during radiation delivery.

Computer-based conformal RT planning techniques, including three-dimensional conformal (3DCRT) and intensity-modulated radiation therapy (IMRT), are designed to maximize the tumor dose while sparing the normal organs surrounding the target, in order to minimize radiation side effects. 3DCRT deliver several uniform fluence beams through one opening field which is shaped by a stable multi-leaf collimator (MLC) (15). IMRT is an advanced radiation delivery technique with use of non-uniform fluence beams, which creates a heterogeneous dose distribution for each beam by using a dynamic MLC (7, 16). IMRT plans can provide superior dosimetric normal tissue sparing compared to 3DCRT. IMRT reduced eyes toxicity in a previous study of 31 intranasal tumor dogs delivered a definitive dose of 42 Gy (16). However, the comparison of the outcome and radiation side effects between 3DCRT and IMRT in a relatively low total dose palliative-intent radiation protocol has not been studied. The aim of this retrospective study was to compare the occurrence of radiation side effects and treatment outcomes in dogs with intranasal tumors treated with a total dose of 20 Gy delivered in 5 daily 4 Gy fractions using 3DCRT or IMRT plans.

## Materials and methods

### Criteria for selection of cases

This study was retrospective in design. The Purdue University Veterinary Teaching Hospital (PUVTH) Radiation Oncology service medical records were reviewed for dogs with intranasal tumors treated with a 4 Gy × 5 daily fraction radiation therapy protocol between March 2010 and December 2017. Inclusion criteria were defined as dogs with a histopathologic diagnosis of malignant intranasal tumor treated with a total dose of 20 Gy delivered in 5 daily 4 Gy fractions with a computer-based 3DCRT or IMRT plan.

### Patient data

Information recorded included patient signalment (breed, age at diagnosis, gender, and weight), medical history, clinical signs, cancer staging results (complete blood count, chemistry panel, abdominal radiography or ultrasound, thoracic radiography or CT, and head CT scan), histopathologic diagnosis, radiation therapy treatment plan details, clinical responses (improvement in clinical signs), radiation side effects, time to local progression (TTLP), PFS, and OS. Follow-up information was obtained from the medical records and by contacting referring veterinarians. TTLP was defined the number of days from the start of radiation therapy to the day of documented tumor local progression in dogs that later dies of tumor-related cause. PFS was the number of days from the start date of radiation therapy to the date of disease progression (local, metastasis or tumor related death). The dogs alive at the end of study, lost to follow-up, or that died without evidence of tumor progression were censored in TTLP and PFS analysis. OS was calculated from the start date of radiation therapy to the date of death. Endpoints were assigned as follows: “Dead” if the dog had died prior to data collection and “Censored” if the dog was known to be alive or with an unknown status (i.e., lost-to-follow-up) in its latest available record.

### Clinical tumor staging

The Adams modified CT staging method was used to assign tumor stage based on radiation therapy planning CT findings reported by a board-certified radiologist in the medical record (2). T1 was tumor confined to one nasal passage or frontal sinus with no bony involvement. T2 was tumor involving bone with no orbital, subcutaneous, submucosal, or nasopharyngeal mass. T3 was tumor involving the orbit or presence of a subcutaneous or submucosal mass. T4 was tumor causing cribriform plate destruction.

### Radiation therapy

All patients were anesthetized and positioned in a thermoplastic mask on an indexable frame (Uniframe Baseplate, Civco Medical Solutions, Orange City, IA) for pre- and post-contrast enhanced CT. Radiation treatment plans were made on pre-contrast CT images using treatment planning software (Varian Eclipse v. 8.9 or 11.0, Varian Medical Systems, Palo Alto, CA). Gross tumor volume (GTV), planning target volume (PTV), and critical normal structures were contoured on the pre-contrast CT. The GTV was delineated using co-registered contrast-enhanced CT images and included the contrast enhancing part of the tumor inside the nasal cavity, frontal sinus and the gross mass extending outside of the nasal cavity. The PTV included a 5- to 10-mm isotropic expansion from the GTV. The goal of planning was for 95% of the PTV to receive ≥95% of the prescribed dose. Critical normal structures were contoured and avoided; defined organs at risk included brain and eyes (left and right contoured separately).

All patients were treated using photons from a 6MV linear accelerator (Varian 6EX, Varian Medical Systems, Inc. Palo Alto, CA) with a 120 leaf multi-leaf collimator (Millennium 120 MLC, Varian Medical Systems, Palo Alto, CA) at an output rate of 400 MU/min. Radiation therapy delivery details were documented in the record and verify system (Aria, Varian Medical Systems, Palo Alto, CA). The IMRT treatment plans were verified using a diode array (Map Check 2, Sun Nuclear Corporation, Melbourne, FL) and the planned X-ray fluence was compared to the machine output X-ray fluence for each plan prior to treatment. The absolute dose at each point was measured using 3.0% absolute dose difference and 3.0 mm distance to agreement. The plan was verified for therapy when >95% of all points matched. Five fractional doses of 4 Gy were delivered to the PTV on 5 consecutive weekdays. Each patient was anesthetized for each fraction. Premedication and induction protocols were selected by a board-certified anesthesiologist based on the patient's clinical status. Anesthesia was maintained with sevoflurane gas in all dogs. All the dogs were positioned in an individualized immobilization device (thermoplastic mask and dental mold) which was made during CT. Orthogonal portal radiographs (dorsoventral and lateral) were taken for position verification of the patients.

### Radiation side effects evaluation

Radiation toxicosis scores based on the Veterinary Radiation Therapy Oncology Group toxicity scoring system (17) were reported in the medical records. Acute radiation side effects were defined as those occurring <90 days after treatment; late radiation side effects were defined as those occurring more than 90 days after treatment.

### Statistical analysis

The commercial software Stata 15.0 (Stata Corp LLC, College Station, Texas, USA) was used to perform the statistical analysis. Statistical analysis was performed by a statistician (H.Y.W.). Chi-squared tests or Fisher's exact tests (if one or more expected counts <5) were used to compare the distribution of the categorical variables, including sex, tumor type, tumor stage, and radiation side effects, between 3DCRT and IMRT groups. T-test was used to compare age and weight of the patients between the two treatment groups, respectively. Median TTLP, PFS and ST were estimated using the Kaplan-Meier method, and the survival curves were compared using log-rank tests. *P-*value <0.05 was considered statistically significant.

## Results

### Patient data

Thirty-six dogs met the inclusion criteria. This included 15 males (all castrated) and 21 females (17 spayed), with a median age of 10.1 years (range, 6.9–14.3 years). Median body weight was 24.3 kg (range, 5.2–47.8 kg). The breeds of dogs included mixed breed (*n* = 14), Collie (*n* = 3), Golden Retriever (*n* = 3), Beagle (*n* = 2), English Springer Spaniel (*n* = 2), Labrador Retriever (*n* = 2), West Highland White Terrier (*n* = 2), and one each of the following: Bearded Collie, Cairn Terrier, Shetland Sheepdog, Maltese, Chesapeake Bay Retriever, American Eskimo, Kerry Blue Terrier, and Welsh Corgi.

Twenty-four dogs (67%) were diagnosed with intranasal carcinoma: Eight dogs with adenocarcinoma, four with squamous cell carcinoma, one with transitional cell carcinoma, and 11 dogs with undifferentiated carcinoma. Ten dogs were diagnosed with intranasal sarcoma (28%): Three dogs with chondrosarcoma, three with osteosarcoma, and four dogs with undifferentiated sarcoma. The other two dogs were diagnosed with olfactory neuroblastoma and malignant neoplasia, respectively.

In clinical tumor staging, six dogs (17%) were classified with T1 disease, eight dogs (22%) with T2 disease, twelve dogs (33%) with T3 disease, and ten dogs (28%) with T4 disease (six dogs had tumor extending into brain). Thirty-one dogs had either bilateral or ipsilateral mandibular lymph node fine-needle aspirates evaluated for cancer staging before beginning radiation treatment. The cytologic samples were reviewed by a board-certified veterinary clinical pathologist. At the time of first treatment, no dog had evidence of lymph node metastasis and two dogs had lung nodules, presumed to be metastasis.

### Radiation treatments

All dogs received a total dose of 20 Gy given 5 daily fractions of 4 Gy. No significant differences in the proportion of patient characteristics were identified between 3DCRT and IMRT groups for age (*p* = 0.149), sex (*p* = 0.154), weight (*p* = 0.495), tumor type (*p* = 0.247), and tumor stage (*p* = 0.055). The 3DCRT used two parallel opposed fields (dorsal and ventral for 15 courses; right and left lateral for 1 courses). IMRT was delivered using 5 to 11 field beam arrangements with inverse optimized treatment planning. The median and mean volume of intranasal tumor (GTV) were 42.75 and 54.75 cm^3^, respectively (range between 7.9 and 184.8 cm^3^). The mean values of dosimetric data for 3DCRT and IMRT was summarized in Table 1. Bilateral mandibular and medial retropharyngeal lymph nodes were also treated prophylactically with same protocol to nasal tumor as a separate target volume in 32 dogs. In one dog with unilateral disease only the ipsilateral lymph nodes (mandibular and medial retropharyngeal) were treated. All dogs were treated concurrently with a combination of NSAIDs and opioids and/or gabapentin for pain relief.

**Table 1:** Dose-volume histogram data between 3DCRT and IMRT plans.

	**3DCRT (*n* = 16)**	**IMRT (*n* =20)**
PTV Dmax (cGy)	2,177 ± 70 (2,022–2,269)	2,384 ± 200 (1,828–2,846)
PTV Dmin (cGy)	1,084 ± 470 (123–1,817)	946 ± 487 (310–2,132)
PTV D2% (cGy)	2,131 ± 64 (2,021–2,242)	2,223 ± 185 (1,645–2,688)
PTV D50% (cGy)	2,056 ± 55 (1,949–2,163)	2,067 ± 161 (1,529–2,480)
PTV D98% (cGy)	1,752 ± 232 (1,121–2,018)	1,714 ± 255 (977–2,162)
Brain D10% (cGy)	807 ± 932 (0.1–2,103)	958 ± 417 (27–1,956)
Brain% >10Gy	12.9 ± 15 (0–45.8)	9.2 ± 6 (0–24)
Ipsilateral eye mean dose (cGy)	892 ± 822 (0–2,078)	485 ± 169 (0–749)
Contralateral eye mean dose (cGy)	303 ± 352 (9–1,163)	471 ± 232 (0–840)

Mean ± SD (range).

PTV, Planning target volume; 3DCRT, 3-D conformal radiation therapy; IMRT, intensity-modulated radiation therapy.

### Reirradiation

Five dogs (listed in Table 2) were reirradiated with the same fractionation protocol (5 daily fractions of 4 Gy) after intranasal tumor local progression which were confirmed with head CT scans. Four dogs were irradiated twice and one dog three times. In the four dogs with two courses of RT treatment, two received two courses of 3DCRT plans, two received two courses of IMRT plans. The dog which was irradiated three times received IMRT twice, followed by one 3DCRT plan. For all dogs treated more than once, the additional courses were initiated in a median of 193 days (6.4 months; range: 60–290 days) following completion of the previous course.

**Table 2:** List of the reirradiated dogs with intranasal tumor.

**Dog**	**Histopathology type**	**Radiation plan**	**Time to local progression (days)**	**Time to reirradiation (days)**	**Survival time (days)**
1	Sarcoma	3DCRT	157	178	408^*^
2	Sarcoma	3DCRT	276	290	388
3	Transitional cell carcinoma	IMRT	171	208	314
4	Malignant neoplasia	IMRT	241	252	647
5	Sarcoma	IMRT, 3DCRT	63, 57	87, 60	421

* Lost follow-up.

### Clinical benefit and side effects

Four dogs had no follow-up assessment of clinical signs documented in the medical records. Among the remaining 32 dogs, clinical signs, including epistaxis, nasal congestion, nasal discharge and facial swelling, were reported complete or partial resolution by the owners in 27 dogs (84%) after the initial treatment course. All the five reirradiated dogs had clinical signs resolution. The median time to clinical signs relief was 12 days (range: 1–88 days) after the last fraction was delivered.

All dogs in all 42 treatment courses had acute side effects assessment documented in the medical records. Acute side effects observed included mild skin toxicity (*n* = 9, 21%), oral mucositis (*n* = 7, 17%), conjunctivitis (*n* = 3, 7%), and temporarily decreased lacrimation (*n* = 3, 7%). Acute radiation side effects were observed following 17 courses in 15 dogs. All acute radiation side effects were classified as grade 1 and grade 2 (only one dog, which was treated with 3DCRT, demonstrated grade 2 skin side effects). Of the four dogs receiving two RT courses, one showed grade 1 acute side effects following each of two IMRT courses, and one showed no side effects following either of two IMRT courses. One dog treated with two 3DCRT courses showed grade 1 side effects following only the first course, and one 3DCRT dog only showed grade 1 side effects following the second course. The dog treated with three courses of RT experienced acute side effects in the second and third course of RT. In the first RT course, 13 dogs were observed with acute RT side effects. Of these dogs, 8 were treated 3DCRT plans (8/16, 50%) and 5 with IMRT plans (5/20, 25%). No significant difference in acute RT side effects between the 3DCRT group and the IMRT group (*p* = 0.121). Five dogs survived <90 days, and these were still at risk for late radiation side effects but succumbed before the occurrences of late radiation side effects. One dog developed cataracts in the ipsilateral eye 99 days after one 3DCRT treatment course. No other clinically significant late side effects were reported. Cosmetic side effects of leukotrichia were documented in four dogs (13%).

### Survival times

**Figure 1:**
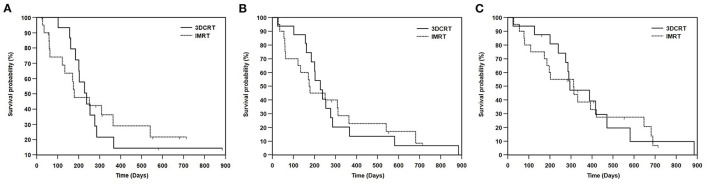
Kaplan-Meier survival curves showing **(A)** TTLP, **(B)** PFS, and **(C)** OS between the dogs treated with 3DCRT or IMRT. The median TTLP for dogs treated with 3DCRT or IMRT was 238 days and 179 days, respectively (*p* = 0.967). The median PFS for dogs treated with 3DCRT or IMRT was 228 and 175 days, respectively (*p* = 0.940). The median OS for 3DCRT or IMRT was 295 days and 312 days, respectively (*p* = 0.787). No significant differences were seen in TTLP, PFS and OS between the 3DCRT and IMRT groups. TTLP, time to local progression; PFS, progression-free survival; OS, overall survival; 3DCRT, 3-D conformal radiation therapy; IMRT, intensity-modulated radiation therapy.

The median follow-up duration was 293 days, with a range between 28 and 885 days. At the time of data analysis, thirty dogs had died or been euthanized (27 due to tumor progression, one due to gastrointestinal lymphoma, and two due to unknown causes). Six dogs were lost to follow-up. For 36 dogs, the median TTLP was 228 days, the median PFS was 201 days, and the median OS was 312 days. The median TTLP for dogs with carcinoma or sarcoma was 238 and 157 days, respectively. The median PFS for dogs with carcinoma or sarcoma was 228 and 157 days, respectively. The median OS for dogs with carcinoma or sarcoma was 312 and 286 days, respectively. The median TTLP for dogs with T1, T2, T3, or T4 disease was 238, 309, 162, and 201 days, respectively. The median PFS for dogs with T1, T2, T3, or T4 disease was 238, 309, 162, and 175 days, respectively. The median OS for dogs with T1, T2, T3, or T4 disease was 471, 314, 286, and 201 days, respectively. The median TTLP for dogs treated with 3DCRT or IMRT was 238 and 179 days, respectively (Figure 1A). The median PFS for dogs treated with 3DCRT or IMRT was 228 and 175 days, respectively (Figure 1B). The median OS for 3DCRT or IMRT was 295 and 312 days, respectively (Figure 1C). Neither TTLP (*p* = 0.967) nor PFS (*p* = 0.940) nor OS (*p* = 0.787) were significantly different between 3DCRT and IMRT groups.

## Discussion

In this study, 36 dogs with intranasal tumors underwent palliative-intent radiation therapy with 20 Gy given in five daily fractions using computer-based plans. The median OS was 10.4 months (312 days), which is comparable to the median ST of between 4.8 and 10.3 months reported previously (11–14, 18). Our median OS results and median PFS (6.7 months) are also similar to the median OS of 10.3 months and median PFS of 5.9 months reported in 18 dogs with intranasal tumors treated with a 4 Gy × 5 daily protocol (13). The common clinical signs in dogs with intranasal tumors include epistaxis, nasal congestion and discharge. The goal of palliative-intent radiation therapy is to relieve clinical signs associated with the tumor and improve quality of life. Palliative-intent radiation therapy is not intended to prolong survival, but improvements in the pet's quality of life may delay an owner's decision to euthanize, resulting in increased survival times. The proportion of improvement in clinical signs in dogs with intranasal tumors following hypofractionated palliative-intent radiation therapy ranges from 83.3 to 100% in previous reports (11–14, 18). In our study, 84 % of dogs had relief of clinical signs a median of 12 days after palliative-intent radiation therapy, which is comparable to the previous reports.

Another goal of palliative-intent radiation therapy is to reduce acute radiation side effects. In the previous prospective study of 18 dogs treated with the same radiation fraction scheme, the planning included manual, CT and MRI plans. Acute side effects were reported in 50% of dogs (13). In our current study, acute radiation side effects occurred in 15 dogs. Eight (50%) dogs exhibited acute side effects in 3DCRT group and five (25%) dogs exhibited acute side effects in IMRT group. To evaluate and compare the biologic effects of different radiation protocols, biologic effective dose (BED) is often used. BED is calculated from the radiation dose per fraction, the number of fractions and the alpha-beta ratio (α/β) of the tissue. The BED for acute responding tissue (α/β = 10) and late responding tissue (α/β = 3) from our 4 Gy × 5 protocol is 28 Gy_10_ and 46.67 Gy_3_, respectively. They are relatively low BEDs for acute and late responding tissues comparing other palliative intent radiation protocols often used in veterinary medicine. Since BED does not take into account the dose frequency and intensity, only comparing BED of daily protocol with weekly protocol might not be fair. All of the acute side effects observed in our study were mild and acceptable with a low rate of occurrence. Most of the late radiation side effects were cosmetic. Clinically significant late effects were observed in only one dog, which developed a cataract following 3DCRT. The mean radiation doses to the eye globes and the lens were 18 Gy and 20 Gy, respectively. No clinically significant late side effects were observed in dogs treated with IMRT in this study.

3DCRT refers to treatments that are based on 3D anatomic information, such as CT images, with treatment fields that conform as closely as possible to the target volume to reduce the radiation dose applied to normal tissue (15). The acute side effects in dogs with intranasal tumors treated with 3DCRT in our study were mild and manageable, and resolved within a few weeks after the completion of radiation therapy. IMRT is a radiation delivery technique that uses beams of non-uniform fluences, which is accomplished by constructing simulated beams and then creating a heterogeneous dose distribution for each beam by using a dynamic multi-leaf collimator which can be adjusted to the irregularly shaped target volumes with high precision while minimizing the radiation delivered to the surrounding healthy tissue and critical structures such as brain, eyes, lacrimal glands, optic nerves, optic chiasm, skin and hard palate mucosa in intranasal tumor cases (7, 16, 19). IMRT plans can provide dosimetric normal tissue sparing and improved tumor coverage compared to 3DCRT for intranasal tumors. Hence IMRT is superior to 3DCRT for maximizing tumor dose while minimizing the dose to surrounding normal tissues. In a previous published study of 31 intranasal tumor dogs treated with IMRT delivered a total dose of 42 Gy, IMRT reduced dose delivered to eyes and resulted in bilateral ocular sparing comparing with conventional 2D plans (16). Although the radiation side effects in our results tended to appear less frequently in dogs treated with IMRT (25%) than either 3DCRT (50%) or in previous published manual/CT plans (50%) (13), there was no statistical difference in the side effects for dogs treated with this relatively low dose palliative-intent protocol.

Five dogs were treated with additional courses using the same palliative-intent RT protocol following tumor progression. Four dogs received two courses (two sarcomas, one transitional carcinoma and one malignant neoplasia). The interval between the first course and the second course ranged between 178 and 290 days (median of 230 days). The dog treated three times was diagnosed with sarcoma. The intervals between the first course and the second course and between the second course and the third course were 87 and 60 days, respectively. The median PFS after the second course was 103 days for the five dogs that received repeated palliative-intent radiation therapy (vs. a median PFS of 201 days for all dogs). Several previous studies also documented that the PFS tended to be shorter for re-irradiation (20, 21).

Limitations of this study include relatively small sample size with low statistical power and the nature of any retrospective study. The results of this study rely on the information in medical records, which may not be complete. Not all dogs returned to our hospital for recommended follow up examinations, hence the effectiveness of palliation, presence of radiation side effects, and local tumor progression were not evaluable at all timepoints as recommended. In addition, as with many studies of nasal tumors in veterinary patients, in most cases local tumor progression was inferred from clinical signs progression rather than based on objective information such as CT scans and biopsies. This presumption could result in misestimation of local tumor progression and PFS. The result of OS may not reflect the goal of palliative-intent protocol, which is to improve quality of life by relieving the clinical signs caused by tumors. The improvement of clinical signs may delay an owner's decision to euthanize (10).

In conclusion, our results support the effectiveness of this protocol for palliation of dogs with intranasal tumors. Significant differences in OS or PFS were not shown between 3DCRT and IMRT techniques. Although the acute radiation side effects observed in this study occurred less frequently in dogs treated with IMRT compared with dogs treated with 3DCRT, these were not statistically different, suggesting that 3DCRT is still useful in this setting. Both 3DCRT and IMRT delivery technique yielded acceptable radiation toxicity in all dogs treated with palliative-intent radiation therapy given in five 4 Gy fractions delivered daily for a total dose of 20 Gy.

## Data availability statement

The original contributions presented in the study are included in the article/supplementary material, further inquiries can be directed to the corresponding author.

## Ethics statement

Ethical review and approval was not required for the animal study because it was a retrospective study. Written informed consent was obtained from the owners for the participation of their animals in this study.

## Author contributions

D-RF and JP conceived the study, designed study design, analyzed the data, and wrote the paper with support of all authors. D-RF, CM, and IV collected and provided the relevant data. IV and JP edited and revised manuscript. H-YW performed the statistical analysis. All authors reviewed and approved the final submitted manuscript.
